# Biosecurity Levels and Farm Characteristics of African Swine Fever Outbreak and Unaffected Farms in Estonia—What Can Be Learned from Them?

**DOI:** 10.3390/ani12010068

**Published:** 2021-12-29

**Authors:** Arvo Viltrop, Kaari Reimus, Tarmo Niine, Kerli Mõtus

**Affiliations:** Institute of Veterinary Medicine and Animal Sciences, Estonian University of Life Sciences, 51006 Tartu, Estonia; kaari.reimus@emu.ee (K.R.); Tarmo.Niine@emu.ee (T.N.); kerli.motus@emu.ee (K.M.)

**Keywords:** disease control, outbreak investigation, biosecurity scoring, risk factor

## Abstract

**Simple Summary:**

Biosecurity breaches have been shown to play a major role in the introduction and spread of African swine fever (ASF) in domestic pig populations. The aim of the study was to describe the biosecurity levels and management practices of ASF outbreak and uninfected herds and to identify potential risk factors for ASF introduction. The biosecurity score of ASF outbreak herds was significantly lower compared to uninfected herds. This may reflect general improvement in the application of biosecurity measures over time in Estonian pig farms as the data on uninfected herds were collected later, at a time when intensified official controls may have had their effect. Larger herds were more at risk of being ‘outbreak herds’ compared to smaller herds. The biosecurity parameters significantly associated with ‘outbreak herd’ status in statistical analysis were mostly related to indirect contacts with the outside farm environment. The biosecurity barriers applied in Estonian pig farms have not been sufficient to avoid the introduction of ASF and need critical evaluation and improvement. Reduction of all contacts between the farm and the external environment should be emphasized in a situation where ASF is circulating in wild boar populations close to pig farms.

**Abstract:**

Risk factors related to external biosecurity have been considered to play a major role in the introduction and spread of African swine fever (ASF) in domestic pig populations. The aim of the study was to describe the biosecurity levels and management practices of ASF outbreak and uninfected herds and to identify potential risk factors for ASF introduction. Data collected from the outbreak herds during outbreak investigations and from the randomly selected uninfected herds were analyzed. The biosecurity score in ASF outbreak herds was significantly lower compared to uninfected herds. However, this may reflect general improvement in the application of biosecurity measures in pig farms over time as the data on uninfected herds were collected later, at a time when intensified official controls may have had their effect. Larger herds were more at risk of being outbreak herds compared to smaller herds. The biosecurity parameters significantly associated with the outbreak herd status in multiple correspondence analysis were mostly related to indirect contacts with the outside farm environment. The biosecurity barriers applied in Estonian pig farms have not been sufficient to avoid ASF introduction and need critical evaluation and improvement. Reduction of all contacts between the farm and the external environment should be emphasized in a situation where ASF is circulating in wild boar populations close to pig farms.

## 1. Introduction

Application of biosecurity measures is considered, and has been proven to be, a primary tool to prevent the introduction and spread of infections in animal populations. In the case of African swine fever (ASF), many risk factors related to biosecurity have been shown to play a major role in spreading the disease and maintaining it in domestic pig populations. The most prominent risk factors are swill feeding and direct contacts between susceptible and infectious pigs (e.g., uncontrolled herd-to-herd movement of pigs) [1]. However, there are few published analytical studies dealing with herd-level risk factors for ASF introduction to domestic pig herds particularly in Europe. Following a literature search of the PubMed.gov database, seven publications from the period 1994 to 2021 could be found where the results of analytical studies (cross sectional, case-control or cohort) on herd-level risk factors for ASF introduction were described (two originating from Nigeria [2,3], one from Uganda [4], one from Kenya [5], one from Italy (Sardinia) [6], one from Estonia [7] and one from Romania [8]). The present understanding of ASF preventive measures largely relies on knowledge about transmission routes of the virus and what measures (actions or processes) could restrict virus transmission, rather than on knowledge of the actual effects of concrete applied measures (such as disinfection of wheels of a vehicle at the farm entrance) in different economic, social and farming practice contexts. This is particularly relevant for biosecurity measures directed against various indirect transmission routes. Quantification of the effectiveness of biosecurity measures would help to define those of particular importance that could save resources for farmers and increase the effectiveness of disease control in general.

Epidemiological investigations in ASF outbreak herds (*n* = 27) from 2015 to 2017 in Estonia revealed that the most probable mode of transmission of the infection into the herds was indirect transmission by contaminated vehicles, humans or other fomites. Biosecurity breaches were identified in all outbreak herds. In some farms with high-level biosecurity measures only minor violations of biosecurity practices may have been the reason for incursion of the virus [7]. Most of the outbreaks occurred in herds with significant biosecurity gaps, such as missing or non-functional physical and/or disinfection barriers to the farm territory, leading to the intuitive conclusion that a low level of external biosecurity (i.e., measures to prevent introduction of a disease) is a main risk factor for the introduction of ASF. However, in general, larger farms, assumed to have a higher level of biosecurity, had a significantly higher risk of contracting the infection (hazard of ASF outbreak HR = 4.22, 95% CI = 1.36–13.14 among herds with 101–1000 pigs compared to farms with <10 pigs) in Estonia [7]. However, a systematic assessment of biosecurity levels in non-infected farms was not undertaken, meaning it was not possible to identify if the average biosecurity level of infected herds truly differed from uninfected herds. In the beginning of 2017, the Estonian Veterinary and Food Board (the national veterinary authority) initiated an external audit of biosecurity measures against ASF in Estonian pig farms to identify any shortcomings and to harmonize the inspection of farms. In addition, a general overview of pig farm biosecurity levels regarding ASF could be obtained. The assessment was requested by an expert group of the Estonian University of Life Sciences (EMU).

Various scoring systems have been used for the quantification of biosecurity levels of pig farms in different countries [9]. Usually, weighting factors are applied to reflect the relative importance of different transmission routes of pathogens and biosecurity measures. These weighting factors are set based on expert opinion [9]. The expert knowledge elicitation methods applied vary. For Biocheck.UGent™ developed by Ghent University [10], the method developed by Gore was used to quantify the expert panel opinions [11]. In other systems, Delfi [12] and analytic hierarchy process (AHP) have been applied [13,14]. Some systems have been developed for specific diseases, such as porcine respiratory and reproductive syndrome [15,16], or are non-disease-specific, such as Biocheck.UGent™ [10]. Recently a self-assessment tool for pig farm biosecurity regarding ASF has been developed in Germany [12,17]. This tool could also be used for external assessments. Nevertheless, according to the terms defined by the proposers the assessment in Estonia had to be based on biosecurity requirements listed in the national legislation. Therefore, the scoring systems available in the literature were not directly applicable in this study and a new one had to be developed.

The aim of the present paper is to describe the biosecurity levels in outbreak and in randomly selected uninfected herds utilizing the data collected during outbreak investigations and farm inspections. In addition, some parameters related to farm management of infected and uninfected farms are described. Due to the time shift between collection of the information from outbreak and uninfected herds, as well as some inconsistency in data collection, it is not possible to make a full comparison of these two groups. However, these observations could form a basis for raising hypotheses about potential risk factors for ASF introduction into pig herds that could be further investigated.

## 2. Materials and Methods

The group of outbreak herds in this study consisted of all ASF-infected herds detected in Estonia from 2015 to 2017 (*n* = 27).

The data from the 2015 outbreak herds (*n* = 18) were extracted from outbreak investigation protocols compiled by the veterinary officers conducting the epidemiological investigations. In addition, the official veterinarians involved, and the farm owners or personnel of outbreak herds, were interviewed face to face or by telephone by the authors during the autumn of 2015 to collect additional information.

In 2016 and 2017, the data on outbreak herds (*n* = 6 and *n* = 3, respectively) were collected by observations and face-to-face interviews during the outbreak investigations conducted by the authors on site.

The biosecurity assessment of uninfected herds was conducted from January to April 2017. The number of uninfected herds to be assessed was determined by available time and human resources. The assessment had to be completed within four months by the expert group of four people with other duties beside this task. Based on this, the target was initially set to visit 30 farms. However, in two instances, two farms selected appeared to belong to the same owner and were located in close proximity (on the same property) and were therefore considered as one epidemiological unit in the analysis. Thus, the final number of visited and assessed uninfected farms (epidemiological units) was 28.

The uninfected herds were selected randomly from the list of all herds in the national animal register using the random number generator in Microsoft Office Excel (MS Excel). The selected herds were located in 11 out of 15 counties of Estonia. Herds from four counties (Hiiumaa, Raplamaa, Ida-Virumaa and Järvamaa) were excluded by the random selection process. The number of herds in these counties was also the smallest (less than 10 herds per county).

One of the herds in the uninfected group contracted ASF in summer 2017 and was moved to the group of outbreak herds in the analysis. One of the 27 reported outbreak herds proved to be uninfected in follow-up studies [7] and was included in the uninfected group. Thus, the final number of outbreak and uninfected herds for this study was 26 and 28 herds, respectively.

The assessment of external biosecurity was performed using the same evaluation criteria (listed in Table 1) in both groups. The assessment was performed by the same expert group (the authors), who performed the ASF outbreak investigations in 2016 and 2017. All biosecurity requirements listed in national legislation were included in the assessment as evaluation criteria. The final evaluation (scoring) of the biosecurity levels of the outbreak and uninfected herds, based on the collected information, was performed by the authors in summer 2018.

In addition to biosecurity parameters (BSP) listed in Table 1, ‘safety of animal movements’, ‘swill feeding’ and ‘rodent and insect control’ formed part of the evaluation of biosecurity, but were omitted from the final scoring for the following reasons: no herd-to-herd transmission of ASF due to animal movement was observed in Estonia [7], there was lack of true evidence of feeding of swill, all herds applied some kind of rodent control, and, for some outbreak herds, information on insect control was missing.

The BSPs used and the scoring system are presented in Table 1. The scale for every BSP (binary or categorical and number of categories) was decided by the research group in panel discussion prior to scoring. Categories were used for BSPs where information for more detailed evaluation was available and the binary assessment would have caused loss of information. The number of categories used for each BSP was based on a subjective consensus decision of the research group.

BSPs were weighted according to importance using an analytic hierarchy process (AHP) [18], applying MS Excel spreadsheet-based AHP software developed by Goepel [19]. The applied method is described in detail by Zang and collaborators [13]. Briefly, the relative importance of the BSPs was assessed individually by every member of the expert panel. The BSPs were evaluated pairwise, semi-quantitatively, by estimating the magnitude of the preference of the alternative on a scale from 1 to 9, where 1 denotes equal importance of two alternatives and 9 extreme importance of one alternative compared to another. Based on pairwise assessments, the relative weight of every parameter was generated on a proportional scale from 0 to 1 (alternatively, 0 to 100%) where higher value represents higher importance. The individual weights of the experts were averaged, and the summary weights of the group were generated by the software algorithm.

The averaged weights of the expert group were used to calculate the score for every assessment criterion by multiplying the score with the weight value and dividing the result by 10. For disinfection barriers at the gate and at the building (criteria 6, 7 and 8 in Table 1), the score was first multiplied by the functionality score (7.1 and 8.1, respectively), and thereafter, with the weight value. For direct contact with other animal species, the average score for criteria 2 and 3 was included in the total score. For disinfection barriers at the farm gate, the average score for people (criterion 6) and vehicles (criterion 7) was included in the calculation of the total.

The final biosecurity score (BSS) for every farm was calculated as the sum of individual weighted scores of every parameter. Higher score indicates a higher biosecurity level of the farm.

In addition to BSPs, information on possible farm-management-related risk factors was collected. Exposure to these risk factors was compared between outbreak and uninfected herds.

### Statistical Analysis

Univariable logistic regression analysis was used to assess the potential associations between the herd infection status and the biosecurity score as well as the studied risk factors. STATA 17 software [20] was used for analysis.

Multiple correspondence analysis (MCA) was conducted to demonstrate potential associations between the variables used for biosecurity scoring and farm outbreak/uninfected status. A two-dimensional symmetric map presenting the solution of two-way interactions of the variable categories was created including all 14 variables used for composing a herd’s BSS (Table 1). These variables were included as active variables determining the solution space and herd outbreak/uninfected status was projected as a supplementary illustrative variable not influencing the geometric orientation of the axes. The centre of the plot coincides with the average of the data set. The farther are the categories from the centre the more they deviate from the average. A higher correlation exists between variables positioned closer in the solution of the MCA. Test values, being the standardized coordinates with mean 0 and variance 1, were used to interpret the significance of the variable category with a threshold of 1.96 corresponding to a *p*-value 0.05 [21,22]. XLSTAT 1 March 2019 [23] was used for conducting the MCA and to perform descriptive statistics for the data.

The distribution map of study herds was developed with the STATA 17 [20] software package “spmap” [24]. The Estonian Land Board map of counties (before public administration reform) was used as a base map [25].

## 3. Results

Distribution of outbreak and uninfected herds between herd size categories and production types are presented in Table 2.

**Figure 1:**
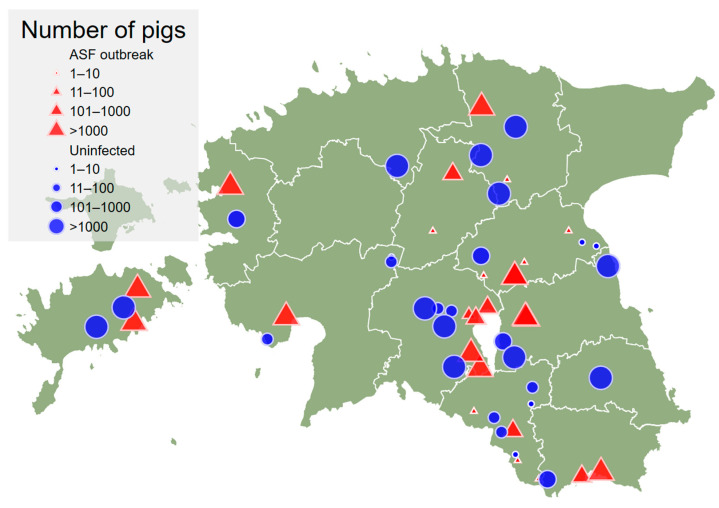
Location of the African swine fever outbreak herds (red triangles; *n* = 26) and uninfected herds (blue circles; *n* = 28) in Estonia. The size of the symbol indicates the size category of the herd based on total number of pigs in the herd.

The map with geographic distribution of outbreak and uninfected herds is presented in Figure 1.

**Figure 2:**
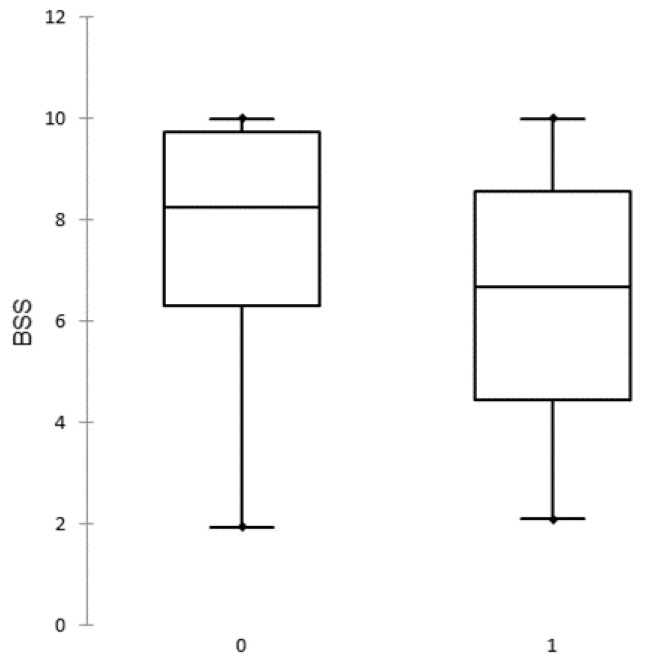
Distribution of biosecurity scores (BSS) in uninfected (0) and African swine fever outbreak (1) herds.

The BSS of all study herds ranged between 1.95 and 10.00 points with a median of 7.70. The frequency distributions of BSS of outbreak and uninfected herds are presented in Figure 2.

The BSS are close to normally distributed in outbreak herds whereas in uninfected herds the distribution is negatively skewed (there are more higher values).

The summary statistics of BSS in outbreak and uninfected herds is presented in Table 3.

The outbreak/uninfected status of the herd was significantly associated with the BSS of the herd (*p* = 0.03) in univariable logistic regression analysis. Production-related parameters had no significant effect on herds’ outbreak/uninfected status.

**Figure 3:**
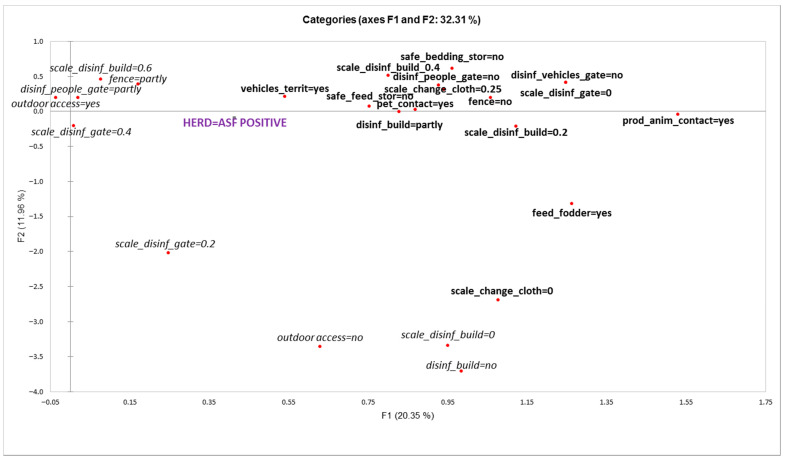
Graphical display of multiple correspondence analysis for variables of biosecurity components associated with herd “outbreak” status (variable categories presented in bold are significantly associated with farm “outbreak” status).

According to the results of the MCA analysis, herd outbreak/uninfected status was significantly associated with the first axis (F1) explaining 20.35% of the total variance (Figure 3). Variable categories that were significantly associated with F1 and herd ’outbreak’ status were the following (variable category presented in MCA plot, Figure 3 and test-values in brackets):

- Other vehicles on territory (vehicles_territ = yes; 4.083)
- Pets in contact with pigs (pet_contact = yes; 4.102)
- Other production animals in contact with pigs (prod_anim_cont = yes; 4.296)
- Feeding freshly cut fodder (feed_fodder = yes; 4.108)
- Storing feed in bins or piles (safe_feed_stor = no; 4.900)
- Unsafe storage of bedding materials (safe_bedding_stor = no; 2.916)
- No fence (fence = no; 4.775)
- No disinfection of people at the entrance of the territory (disinf_people_gate = no; 5.042)
- No disinfection of vehicles at the entrance of the territory (disinf_vehicles_gate = no; 6.152)
- Dysfunctional disinfection at the entrance of the territory (scale_disinf_gate = 0; 6.152)
- Disinfection barrier at every entrance of the building is partly present (disinf_build = partly; 3.047)
- Poor functionality of disinfection barrier at the entrance to the building:
  ○ Score value 0.2 (scale_disinf_build = 0.2; 3.121)
  ○ Score value 0.4 (scale_disinf_build = 0.4; 2.606)
- Low safety of changing clothes before entering the pig facility:
  ○ Score value 0 (scale_change_cloth = 0; 2.218)
  ○ Score value 0.25 (scale_change_cloth = 0.25; 5.171)

## 4. Discussion

Data collection for this study was conducted over a three-year period from 2015 to 2017. The majority of the outbreak herds were from years 2015 and 2016 (three from 2017). All but one uninfected herd were visited in 2017. This must be taken into consideration when interpreting the results as since 2015 the biosecurity requirements for pig farms were tightened in Estonia and official inspections of farms became more frequent (at least twice a year). These measures have probably caused improvements in the biosecurity of farms overall.

From outbreak and uninfected herds, data were collected by the same group of experts following the same study protocol. The questionnaire was discussed thoroughly by the team members before the assessments to ensure uniformity in evaluations. However, data collection for the 2015 outbreak herds was conducted retrospectively using the outbreak investigation protocols and questioning the people involved in outbreak management. This may have had some impact on evaluations of a more subjective nature (such as the functionality of disinfection barriers) or some recall bias. Nevertheless, in general, we were able to retrieve the same information about 2015 outbreak-farms as during the farm visits conducted later. The interpretation of the collected information was uniform for all herds which reduced the effect of these differences in data collection.

The uninfected herds were selected randomly from the whole population of Estonian pig herds. The geographical distribution of uninfected herds coincided with the geographical distribution of outbreak herds well (Figure 1). Just one uninfected herd occurred in a county (Harjumaa) where outbreaks were not observed.

The biosecurity measures assessed in this study were mostly prescribed by national legislation [26] and included generally recognized physical elements of external biosecurity, such as fencing and other physical barriers, disinfection barriers and changing rooms, as well as storage conditions of feed and bedding materials, etc. The assessment was conducted taking into account the specific hazards associated with the spread of ASF among wild boar and the need to avoid transmission (mainly indirect) of the virus from that source. Thus, some very specific requirements were introduced in the legislation (and the assessment), such as a ban on having outdoor access for pigs and a ban on feeding fresh fodder.

The biosecurity scoring was mainly based on counting the biosecurity elements present. In the case of a single visit to the farm, it was difficult to evaluate compliance with the biosecurity requirements by the farm personnel and visitors. Nevertheless, it was possible to evaluate, to some extent, the functionality of the disinfection barriers based on observations on site and methods used for disinfection, as well as the effectiveness of some biosecurity procedures, such as entering of people and vehicles into the pig-keeping rooms.

To reflect the greater importance of some biosecurity measures and elements compared to others, the weights were incorporated into the calculation of the BSS using an analytic hierarchy process to evaluate the importance of different biosecurity measures. However, using the weighted scores compared to unweighted scores had only a little effect on the biosecurity level estimates of individual farms (i.e., only a few farms ‘changed’ their position in the ordered list and in relation to the median of all study herds). The difference between the average scores of outbreak and uninfected groups was not significantly modified by applying weighted scores (data not shown).

Multiple correspondence analysis was used to find management-related risk factors associated with herd outbreak/uninfected status. This method was chosen due to it being less sensitive to multicollinearity and because of a relatively small number of observations with a high number of explanatory variables. Nonetheless, since only two-way associations were analyzed, the effect of the associations was not adjusted for the influence of other factors, nor could interactions be tested [21,22]. Therefore, the presented associations should be considered as potentially relevant.

The results of the univariable analysis showed that the biosecurity level in uninfected herds was significantly higher than in outbreak herds. However, as mentioned above, this difference may reflect the improvement of biosecurity level in Estonian pig farms over the three-year period. The average BSS of the year 2017 outbreak herds was 8.8 being higher than the mean (7.9) of all uninfected herds. There is also some indirect evidence that may indicate that present biosecurity measures applied (or as applied) in pig farms might have not been sufficient to avoid ASF outbreaks in Estonian pig farms. First, the herd incidence of ASF outbreaks in Estonia did not change significantly over the years 2015–2017 being in the range of 2.1–2.6 outbreaks per 100 herd-years [7] although the biosecurity requirements of farms were tightened and official checks made more frequent. Secondly, the herd incidence among larger herds was significantly higher compared to smaller herds in Estonia [7]. It is assumed, as well as demonstrated, that small-holders, in general, are less capable of applying proper biosecurity measures [27,28,29]. However, in practice larger herds appeared to be at higher risk of contracting the ASF in Estonia. Further, in an ASF case-control study conducted in Romania, the case herds appeared to be significantly larger in the study group of backyard farms whereas, among commercial farms, the herd size was not a significant factor [8]. However, the incidence of ASF outbreaks in different herd size groups (e.g., commercial vs backyard) could not be compared in this study. Nevertheless, among backyard farms there was a tendency for case farms to have had more external visitors and more vehicles entering the farm. These are factors presumably associated with herd size and could be considered as a reason for the higher frequency of virus introductions. An analysis of ASF serological surveillance data conducted in Sardinia (Italy) in the late 1990s [6] demonstrated that the probability of a herd containing seropositive pigs was negatively associated with the level of confinement of the pig keeping system (outdoor vs. semi-outdoor vs. indoor), but positively associated with the number of pigs sampled from the herd reflecting the herd size (analyzed with a multivariable model). These results demonstrate that, on the one hand the level of biosecurity of the farm is important in reducing the risk of disease introduction, but at the same time larger herd size appears to be a factor that increases the risk of virus introduction. This needs to be accounted for when the efficacy of biosecurity measures is evaluated in observational studies. There is also an indication that larger farms must apply more stringent biosecurity rules to keep the risk of disease introduction low.

Due to the small sample size of this study, we were not able to reliably compare the biosecurity levels of outbreak and uninfected herds in different herd size categories. Nevertheless, the average BSS was higher in larger herds compared to smaller ones (data not shown).

If improvements in the meeting of formal biosecurity requirements have not reduced the risk of outbreaks, the reason may be that the applied measures are not effective enough or they are not properly applied (i.e., compliance to procedures). As an example, Estonian pig farmers have not been able (as it is economically and technically not feasible) to introduce a washing step before disinfection of vehicles entering the farm territory. The spraying of vehicles is not compulsory and often only the wheels are disinfected by passing over a disinfection bath or mat. This may be insufficient to avoid the carriage of contaminant through the disinfection barrier even if proper waiting time is adhered to. The second reason may be that with applied biosecurity measures not all transmission routes are controlled. Due to the strong seasonal pattern in the occurrence of ASF outbreaks in domestic pigs (the summer season) the role of blood-sucking insects in virus transmission has been discussed [30,31,32]. In Estonia, pig stables are not insect-proof and this has not been considered as necessary to date. Other measures to control insects (mainly flies) within the pig facility have been applied in most farms but these are not directed at avoiding incursion of insects from outside.

The results of the multiple correspondence analysis (MCA) revealed three groups of factors potentially associated with the infection status of a herd: First, factors related to physical and disinfection barriers (e.g., lack of fencing and disinfection barriers, poor functionality of barriers). Second, factors related to frequency of contacts to the ‘outside world’ (e.g., the presence of other vehicles than those necessary on the territory of the farm, contacts with other animal species). Third, factors related to storage and use of feed and bedding material. All these factors are mainly associated with indirect transmission routes of the infection and are consistent with the assumed virus introduction routes into outbreak herds identified during the outbreak investigations [7]. This emphasizes once again the effect of frequency of contacts on the probability of transmission—with increase in the number of contacts as potential sources of infection, the chance to experience one ‘effective contact’ will increase given that the probability of transmission per contact will not decrease. Thus, reduction of all contacts between the farm and external environment should be emphasized in a situation where ASF is circulating in wild boar populations close to pig farms.

The MCA analysis did not reveal any significant associations between farm management indicators and the outbreak/uninfected status of herds. However, due to the small sample size of the study, we might not have been able to identify the less significant associations.

It is evident that the biosecurity barriers applied in Estonian pig farms have not been sufficient to avoid ASF introductions. Outbreaks have occurred in farms where the level of biosecurity was evaluated as high. The herd incidence of outbreaks did not change significantly over the years [7] despite biosecurity requirements enforced by the authorities and the frequency of checks being increased. This is an indication that the measures applied to date need critical evaluation and improvement.

## 5. Conclusions

- The biosecurity level in ASF outbreak herds (6.34; SD 2.42) was significantly lower than in uninfected herds (7.86; SD 2.09) in Estonia. However, this may reflect a general improvement in the application of biosecurity measures in pig farms over a three-year period.
- The factors associated with the ‘outbreak’ status of a herd included unsafe contacts with the outside farm environment, such as lack of fencing, lack of or poor disinfection barriers at the entrance of the farm territory for people and vehicles, poor safety procedures for changing clothes before entering the pig facility, contacts with other domestic animals, and entering of unnecessary vehicles on the territory of the farm.
- Another distinctive group of factors associated with outbreak status of the herd related to feed and bedding, such as feeding freshly cut fodder, storing feed in bins or piles, and unsafe storage of bedding materials.

## Figures and Tables

**Table 1:** The evaluation criteria, the scoring system and weights values used in assessing the external biosecurity of pig farms in Estonia.

Criterion	Scoring	Weight Values
1. Outdoor access for pigs	0/1 (yes/no)	17
2. Direct contact with other farm animals	0/1 (yes/no)	13
3. Direct contact with dog or cat	0/1 (yes/no)	13
4. Fencing of the farm territory	0 to 1 (no/partly yes/yes) with 0.5 increment	4
5. Allowing unnecessary vehicles to enter the territory of the farm	0/1 (yes/no)	4
6. Disinfection barrier at the gate of the territory for vehicles	0 to 1 (no/partly yes/yes) with 0.5 increment	5
7. Disinfection barrier at the gate of the territory for people	0 to 1 (no/partly yes/yes) with 0.5 increment	5
7.1 Functionality of the disinfection barrier at the gate	0 (least) to 1 (most) with 0.2 increment	NA *
8. Disinfection barrier upon entry into the building for people and vehicles	0 to 1 (no/partly yes/yes) with 0.5 increment	10
8.1 Functionality of the disinfection barrier at the entrance to the building	0 (least) to 1 (most) with 0.2 increment	NA *
9. Secure change of clothes before entering the pig facility ^1^	0 (least) to 1 (most) with 0.25 increment	12
10. Safe storage of feed (incl. safe water source) ^2^	0 (least) to 1 (most) with 0.5 increment	12
11. Safe storage of litter ^3^	0 (least) to 1 (most) with 0.5 increment	8
12. Feeding of freshly cut fodder to pigs	0/1 (yes/no)	15

* NA—not applied. ^1^ ‘least secure’—no change, ‘most secure’—full change and shower before entering the pig facilities; ^2^ ‘least safe’—in the bin on the floor of a storage room, ‘most safe’—in a closed bunker with automated filling and outlet; ^3^ ‘least safe’—outdoor in an open bin; ‘most safe’—inside of closed room or no use of bedding.

**Table 2:** Distribution of outbreak and uninfected herds between herd size categories (**A**) and production types (**B**).

**A**	**Herd size Categories** **(n Pigs)**	**Outbreak Herds**	**Uninfected Herds**	**Total**
	1–10	8	5	13
	11–100	1	7	8
	101–1000	5	4	9
	>1000	12	12	24
	Total	26	28	54
**B**	**Production Type**	**Outbreak Herds**	**Uninfected Herds**	**Total**
	Farrow to finish	9	12	21
	Fattener	14	13	27
	Breeders	3	3	6
	Total	26	28	54

**Table 3:** Descriptive statistics of biosecurity score values in outbreak and uninfected herds.

Herd Category	Observations	Minimum	Maximum	Median	Mean	SD ^1^
Uninfected herds	28	1.95	10.00	8.26	7.86	2.09
Outbreak herds	26	2.10	10.00	6.68	6.34	2.42

^1^ Standard deviation.

## Data Availability

Restrictions apply to the availability of these data. The owner of data is the Estonian Agriculture and Food Board (former Veterinary and Food Board) and are available from the authors with the permission of Estonian Agriculture and Food Board.
